# Cortical amyloid-beta burden is associated with changes in intracortical myelin in cognitively normal older adults

**DOI:** 10.1038/s41398-023-02420-7

**Published:** 2023-04-06

**Authors:** Marina Fernandez-Alvarez, Mercedes Atienza, Jose L. Cantero

**Affiliations:** 1 Laboratory of Functional Neuroscience, Pablo de Olavide University, Seville, Spain; 2 CIBERNED, Network Center for Biomedical Research in Neurodegenerative Diseases, Madrid, Spain

**Keywords:** Prognostic markers, Molecular neuroscience

## Abstract

Amyloid-beta (Aβ) aggregates and myelin breakdown are among the earliest detrimental effects of Alzheimer’s disease (AD), likely inducing abnormal patterns of neuronal communication within cortical networks. However, human in vivo evidence linking Aβ burden, intracortical myelin, and cortical synchronization is lacking in cognitively normal older individuals. Here, we addressed this question combining ^18^F-Florbetaben-PET imaging, cortical T1-weigthed/T2-weighted (T1w/T2w) ratio maps, and resting-state functional connectivity (rs-FC) in cognitively unimpaired older adults. Results showed that global Aβ burden was both positively and negatively associated with the T1w/T2w ratio in different cortical territories. Affected cortical regions were further associated with abnormal patterns of rs-FC and with subclinical cognitive deficits. Finally, causal mediation analysis revealed that the negative impact of T1w/T2w ratio in left posterior cingulate cortex on processing speed was driven by Aβ burden. Collectively, these findings provide novel insights into the relationship between initial Aβ plaques and intracortical myelin before the onset of cognitive decline, which may contribute to monitor the efficacy of novel disease-modifying strategies in normal elderly individuals at risk for cognitive impairment.

## Introduction

The accumulation and aggregation of amyloid-beta (Aβ) isoforms into senile plaques is the earliest recognizable event in Alzheimer’s disease (AD). These first Aβ deposits precede overt clinical symptoms by decades [1–5] and they supposedly instigate the pathogenic cascade that leads to cognitive decline [6]. This hypothesis is supported by evidence indicating that subtle memory decline begins to appear with subthreshold amyloid PET [7]. Notably, early Aβ deposition initiates in weakly myelinated cortical regions [2], suggesting that Aβ plaques and abnormal cortical myelination are coupled events years before the onset of clinical manifestations.

Converging research has reinforced the link between cerebral Aβ pathology and myelin damage. In this context, in vitro experiments have shown that exposure to Aβ_1–42_ not only impairs the function of rodent oligodendrocytes and oligodendrocyte progenitor cells [8, 9] but also inhibits myelin sheet formation [10]. These findings have also been confirmed in transgenic mouse models that overexpress the amyloid precursor protein (APP) [11–14]. Evidence also supports the hypothesis that myelin breakdown drives Aβ pathology. Accordingly, myelin damage not only precedes Aβ deposition [15, 16] but also promotes plaque formation in mouse models of AD [17]. Regardless of whether myelin alterations precede or result from Aβ deposition, there is lack of evidence supporting associations between cortical Aβ burden and cortical myelin content in cognitively normal older people, which may contribute to monitor the efficacy of novel disease-modifying strategies on early Aβ toxicity in individuals at risk for cognitive decline.

Here, we investigated whether variations in global Aβ load parallel cortical changes in the T1-weigthed/T2-weighted (T1w/T2w) ratio in cognitively unimpaired older adults. The T1w/T2w ratio is considered a measure of the microstructural integrity of the normal-appearing cortical grey matter (GM) [18] able to detect intracortical myelin content [19]. Based on evidence showing that early Aβ pathology initiates in poorly myelinated areas of the human neocortex [2], our prediction was that cortical Aβ burden, as revealed by ^18^F-Florbetaben-PET imaging, would be associated with lower T1w/T2w ratio in late-myelinating cortical regions.

Previous studies have revealed that subtle changes in myelination affect the speed of action potential conduction, thereby influencing neural circuit dynamic and precision in neuronal communication [20–23]. On the basis of this evidence, we hypothesized that cortical regions exhibiting correlations between T1w/T2w ratio and global Aβ load will also show impaired patterns of resting-state functional connectivity (rs-FC) and associations with subclinical cognitive deficits. Given that age-related slowing in cognitive processing speed seems to be mediated by myelin breakdown [24], we expected processing speed to be particularly associated with cortical regions affected by the relationship between T1w/T2w ratio and global Aβ burden, although other cognitive functions may also be similarly affected [25].

## Methods

### Participants

Seventy-four cognitively normal older adults participated in the study (66.5 ± 5.7 years; range: 54–76 years; 51 females). They were recruited from senior citizen’s associations, health-screening programs, and hospital outpatient services. All of them underwent neurological and neuropsychological assessment to discard the presence of objective cognitive impairment and/or dementia. Individuals with medical conditions affecting brain structure or function (e.g., cerebrovascular disease, epilepsy, head trauma, history of neurodevelopmental disease, alcohol abuse, hydrocephalus, and/or intracranial mass) were not included in the study. Participants met the following criteria: i) normal global cognitive status in the Mini-Mental State Examination (scores ≥ 26); ii) normal cognitive performance in the neuropsychological tests relative to appropriate reference values for age and education level; iii) global score of 0 (no dementia) in the Clinical Dementia Rating; iv) functional independence as assessed by the Spanish version of the Interview for Deterioration in Daily Living Activities;[26] v) scores ≤ 5 (no depression) in the short form of the Geriatric Depression Scale;[27] and vi) not be taking medications that affected cognition, sleep, renal and/or hepatic function. All participants gave informed consent to the experimental protocol approved by the Ethical Committee for Clinical Research of the Junta de Andalucía according to the principles outlined in the Declaration of Helsinki. Table 1 contains sample characteristics.

**Table 1:** Demographic and cognitive profile of the study sample.

Age	66.5 ± 5.7
Sex (F/M)	51/23
Education years	12.5 ± 4.9
ApoE4 (yes/no)	16/58
MMSE	29.0 ± 1.2
Memory Binding Test	
Total free recall	15.5 ± 4.4
Total paired recall	24.6 ± 4.2
Total delayed-free recall	16.4 ± 4.9
Total delayed paired recall	24.0 ± 4.6
S-FNAME	32.6 ± 15.2
Boston Naming Test	12.1 ± 2.1
Phonological fluency	15.7 ± 4.4
Semantic fluency	22.0 ± 17.0
Trail Making Test-A	47.0 ± 21.5
Trail Making Test-B	119.5 ± 67.7
Tower of London	319.3 ± 113.2
D2	364.1 ± 94.0
Letter-number sequencing	9.1 ± 2.3
Digit span	14.3 ± 3.2
Symbol digit modalities test	38.5 ± 12.2

Results are expressed as mean ± standard deviation (SD), unless otherwise stated. *F/M* females/males; *MMSE* Mini Mental State Examination; *S-FNAME* Spanish validation of the Face Name Associative Memory Exam.

### Neuropsychological assessment

Subjects were administered with a neuropsychological battery to assess memory, attention, processing speed, and executive function skills such as working memory, inhibition, flexibility and planning. Neuropsychological tests included the Spanish version of the memory binding test (MBT) [28], the face-name associative memory exam (S-FNAME) [29], the D2 test, the letter-number sequencing and the digit span subtests of the Wechsler Adult Intelligence Scale-III, the spatial span subtest of the Wechsler Memory Scale-III, the short form of the Boston naming test (BNT), the semantic and phonological fluency tests based on the “Animal” and letter “P” naming tasks, the symbol digit modalities test, the two forms of the trail making test (TMT-A and TMT-B), and the tower of London (TL). All scores were z transformed. Inverse z-values were used in those cases where higher scores corresponded to worse performance. We further computed the Spearman’s ‘g’ factor as an index of global cognitive function. This analysis was done with R Statistical Software v3.0.1 (R Foundation for Statistical Computing, Vienna, Austria) using the *prcomp* function. We only retained the first component (eigenvalue 8.7), which explained 37.7% of variance in the data.

### MRI acquisition, preprocessing, and generation of T1w/T2w ratio maps

Images were acquired on a 3 T Philips Ingenia MRI scanner using a 32-channel receive-only radio-frequency (RF) head coil and a transmit RF body coil (Philips, Best, Netherlands). The following MRI sequences were acquired in the same session: i) 3D T1-weighted (T1w) magnetization prepared rapid gradient echo (MPRAGE) in the sagittal plane: repetition time (TR)/echo time (TE) = 2600 ms/4.7 ms, flip angle (FA) = 9°, acquisition matrix = 384 × 384, voxel resolution in acquisition = 0.65 mm^3^ isotropic, resulting in 282 slices without gap between adjacent slices; ii) 3D T2w VISTA Turbo Spin Echo scan in the sagittal plane: TR/TE: 2500 ms/251 ms, FA = 90°, acquisition matrix = 384 × 384 mm, voxel resolution in acquisition = 0.65 mm^3^ isotropic, resulting in 282 slices without gap between adjacent slices; and iii) T2w Fast Field Echo images using a blood-oxygen-level-dependent (BOLD) sensitive single-shot echo-planar imaging (EPI) sequence in the axial plane: TR/TE: 2000 ms/30 ms, FA = 80°, acquisition matrix = 80 × 80 mm, voxel resolution in acquisition = 3 mm^3^ isotropic, resulting in 35 slices acquired in posterior to anterior phase-encoding direction with 1 mm of gap between adjacent slices. Pulse and respiratory rates were simultaneously recorded using the scanner’s built-in pulse oximeter placed on the left-hand index finger and a pneumatic respiratory belt strapped around the upper abdomen, respectively. Before starting the acquisition of the EPI sequence, participants were asked to remain still and keep their eyes closed without falling sleep. We acquired 250 EPI scans preceded by 4 dummy volumes to allow time for equilibrium in the spin excitation. To allow for optimal B1 shimming, a B1 calibration scan was applied before starting the EPI sequence. Brain images were visually examined after each MRI sequence; they were repeated if artifacts were identified. All participants underwent the same protocol in the same MRI scanner at the research MRI facility located at Pablo de Olavide University.

T1w scans were preprocessed using Freesurfer v6.0 (https://surfer.nmr.mgh.harvard.edu/). The Freesurfer’s pipeline included brain extraction, automated tissue segmentation, generation of white matter (WM) and pial surfaces, correction of surface topology and inflation, co-registration, and projection of cortical surfaces to a sphere for the purpose of establishing a surface-based coordinate system [30]. Pial surface misplacements and erroneous WM segmentation were manually corrected on a slice-by-slice basis by one experienced technician. T2w images were registered to T1w images with *bbregister* using a trilinear interpolation method and a boundary-based cost function constrained to 6 degrees of freedom [31].

Individual T1w/T2w ratio volumes were sampled at the halfway between the WM and GM surface, resulting in midthickness surface maps of the T1w/T2w ratio. To mitigate contamination of cortical GM intensity by intensities of WM and cerebrospinal fluid (CSF), the tissue fraction effect was corrected in individual T1w/T2w ratio maps using the geometric transfer matrix-derived region-based voxel-wise method implemented in PETsurfer [32]. Finally, individual T1w/T2w ratio maps were projected onto the average cortical surface of the sample, and smoothed using non-linear spherical wavelet-based denoising schemes [33]. All processing steps were visually checked for quality assurance.

### Amyloid PET acquisition and preprocessing

To estimate global Aβ load in the brain, participants were injected with 300 MBq of ^18^F-Florbetaben (FBB, NeuraCeq™, Piramal Pharma) 90 min before image acquisition in a Philips Gemini 16 PET/CT scanner (Philips, Best, Netherlands). PET data were corrected for radioactive decay, dead time, attenuation, and scatter. Cerebral PET images were reconstructed iteratively with an isotropic voxel resolution of 2 mm^3^. Participants underwent a 20-min FBB-PET scan in dynamic mode consisting of four frames of 5 min each. Each frame was inspected for excessive motion. As no excessive head motion was detected in scanned images, the four frames were averaged to create a single static FBB brain image used for quantitative analysis.

Partial volume correction (PVC) of FBB PET images was performed with PetSurfer. For this, we employed the geometric transfer matrix-derived region-based voxel-wise method assuming a uniform 6 mm point spread function. Next, PVC-cortical FBB images were transformed into standardized uptake value ratio (SUVR) using the mean PVC uptake of the cerebellar GM as reference region. Finally, we obtained the global cortical Aβ load for each participant using an FBB composite extracted across 4 large bilateral regions: frontal (orbitofrontal cortex/inferior frontal gyrus/middle frontal gyrus/superior frontal gyrus/frontal pole), cingulate (anterior cingulate/posterior cingulate/isthmus cingulate), parietal (precuneus/inferior parietal cortex/superior parietal cortex/supramarginal gyrus), and lateral temporal (middle temporal/superior temporal gyri) [34].

### Functional MRI preprocessing and rs-FC analysis

The rs-fMRI data were preprocessed using AFNI functions (https://afni.nimh.nih.gov/afni), version AFNI_20.3.01. For each participant, high-frequency spikes were eliminated (*3dDespike*), time-locked cardiac (measured by pulse oximeter) and respiratory motion artifacts on brain BOLD signals were minimized using RETROICOR [35], time differences in slice-acquisition were corrected (*3dTshift*), EPI scans were aligned using rigid body motion correction and the first volume as reference (3*dVolreg*), and aligned EPI scans were co-registered to their corresponding T1w volumes (*align_epi_anat.py;* cost function: lpc+ZZ).

Dynamics were removed provided that more than 5% of voxels exhibited signal intensities that deviated from the median absolute deviation of time series (*3dToutcount*), and/or when the Euclidean norm (*enorm*) threshold exceeded 0.3 mm in head motion. None of the participants showed more than 20% of artifactual dynamics after applying censoring. Simultaneous regression was further applied to minimize the impact of non-neuronal fluctuations on the rs-fMRI signal (*3dTproject*). Nuisance regressors included: i) six head motion parameters (3 translational and 3 rotational) derived from the EPI scan alignment along with their first-order derivatives, ii) time series of mean total WM/CSF signal intensity, and iii) cardiac and respiratory fluctuations plus their derivatives to mitigate effects of extracerebral physiological artifacts on brain BOLD signals.

Preprocessed rs-fMRI scans were projected onto the 5th-order icosahedral tesselation of the average cortical surface. Seeds for rs-FC analyses were obtained from cortical regions showing significant correlations between global Aβ load and T1w/T2w ratio maps. Surface-based rs-FC seed to whole cortex maps were computed using the Fisher’s z-transform of the corresponding Pearson’s correlation coefficients.

### Statistical analysis

We first performed vertex-wise multiple linear regression analyses to evaluate associations between global Aβ load and T1w/T2w ratio maps. These analyses were adjusted by age, sex, education years, body mass index (BMI), insulin resistance estimated with the homeostasis model assessment (HOMA-IR) [36], and metabolic status evaluated as a continuous score [37]. The clusters resulting from this analysis were used as seeds to generate the rs-FC maps aimed at evaluating whether significant and positive rs-FC patterns were moderated by the interaction between global Aβ load and mean T1w/T2w ratio of the region used as seed in the rs-FC map. These models were also adjusted by the above-mentioned covariates.

Vertex-wise regression analyses for cortical T1w/T2w ratio and rs-FC were performed with the SurfStat package (https://www.math.mcgill.ca/keith/surfstat/). Results were corrected for multiple comparisons using a hierarchical statistical model that first controls the family-wise error rate at the cluster level by applying random field theory over smoothed statistical maps (*p*_vertex_ < 0.001, *p*_cluster_ < 0.05), and next controls the false discovery rate at the vertex level within each cluster (*p* < 0.05) over unsmoothed statistical maps [38]. Peaks of clusters that survived correction for multiple comparisons were employed to establish the anatomical location of significant changes using the Desikan-Killiany atlas [39].

After inferential evidence of a main effect, the standardized measure of effect size (i.e., Cohen’s f^2^) was obtained to evaluate the local effect size for a multivariate regression model [40]. To establish the precision of standardized effect sizes, we computed 95% confidence intervals (*CI*_95%_) with the Matlab’s *bootci* function using the normal approximated interval with bootstrapped bias and standard error (*N* = 10.000 bootstrap samples).

For each cortical vertex showing the maximum statistic within each significant cluster, we further applied Bayesian linear regression analyses using JASP, version 0.12.2 (https://jasp-stats.org/). The Bayesian approach allowed us to quantify the evidence for the alternative hypothesis and to overcome the problem of multiple comparisons across contrasts. Bayesian linear regression analyses were based on the Jeffreys-Zellner-Siow prior with an r scale of 0.354 [41]. The strength of the Bayes factor for the model including all covariates of no interest (null model) was compared with the model including the predictor of interest (experimental model) (BF_10_). The classification scheme proposed by Lee and Wagenmakers was employed to interpret the BF_10_ [42]. Clusters were reported as long as the *p* value yielded by the frequentist approach was < 0.05, the standardized size effect was at least small, and the evidence in favor of the alternative hypothesis was at least moderate (BF_10_ ≥ 3).

Next, we evaluated whether cortical regions showing a significant association of global Aβ load with either T1w/T2w ratio or rs-FC accounted for the variability in cognitive function. For this, we first applied the Yeo-Johnson transformation to the cognitive scores to mitigate the detrimental effects of skewedness and heteroscedasticity on different models [43]. We next built three models to assess the main and interaction effects. The first model (null model) only included the covariates of no interest, while the experimental models included either the mean T1w/T2w ratio or the mean rs-FC in a particular cluster as the main regressor of interest. The experimental and null models were compared with an ANOVA by applying both frequentist and Bayesian approaches.

Finally, we assessed whether associations between Aβ-related changes in T1w/T2w ratio and cognitive performance were mediated, at least partially, by global Aβ load. For this, we performed causal mediation analysis using the *mediate* function of the Mediation R package. Unstandardized indirect effects were computed for each of 1000 bootstrapped samples using 95% bias-corrected and accelerated confidence intervals at the 2.5th and 97.5th percentiles.

## Results

### Relationship between global Aβ load and T1w/T2w ratio

**Fig. 1:**
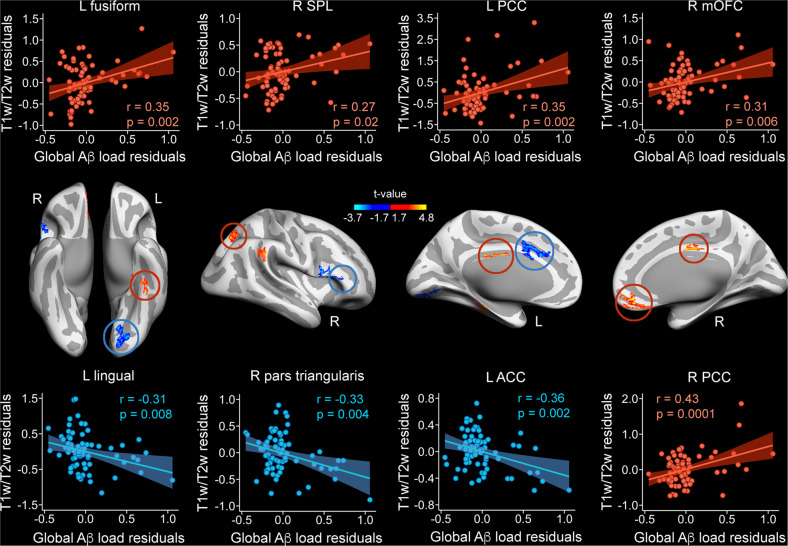
Partial regression plots showing significant correlations between global Aβ load and T1w/T2w ratio. Scatter plots (upper and bottom panels) show positive (red) and negative (blue) correlations in cortical regions marked in red and blue circles, respectively (middle panel). Figure axes show the residuals from the T1w/T2w ratio and the rs-FC. SPL superior parietal lobe, PCC posterior cingulate cortex, mOFC medial orbitofrontal cortex, ACC anterior cingulate cortex. Right: right, L: left.

We first identified those cortical regions where the T1w/T2w ratio varied with global Aβ load. The results of multiple regression analyses are displayed in Fig. 1 and summarized in Table 2. The direction of the relationship was negative in the left anterior cingulate, left lingual gyrus and different areas of the right inferior frontal gyrus, whereas it was positive in the left parahippocampal gyrus, right frontoparietal regions, and posterior cingulate bilaterally. Remarkably, the effect sizes were highest in the right posterior cingulate and left parahippocampal gyrus, two cortical regions showing positive correlations between global Aβ load and T1w/T2w ratio. Bayesian linear regression analyses showed extreme evidence for the positive associations in the right medial orbitofrontal and the posterior cingulate bilaterally (Table 2).

**Table 2:** Cortical regions showing significant correlations between global Aβ load and myelin content.

	MNI	*R*^2^	F_13,60_	$f_{local}^2$	CI_95%_	BF_10_
*Negative correlations*						
L anterior cingulate (*p*_*cluster*_ = 0.002)	−11 18 32	0.13	10.8	0.14	0.008–0.49	26 ^S^
L lingual gyrus (*p*_*cluster*_ = 0.002)	−25–80–9	0.15	12.8	0.10	0.01–0.28	6 ^M^
R pars opercularis (*p*_*cluster*_ = 0.008)	46 10 20	0.16	13.4	0.11	0.004–0.36	6 ^M^
R pars triangularis (*p*_*cluster*_ = 0.02)	52 23 11	0.15	12.9	0.12	0.02–0.45	67^VS^
*Positive correlations*						
L posterior cingulate (*p*_*cluster*_ = 0.01)	−3–8 29	0.19	17.4	0.14	0.005–0.5	12355^E^
L fusiform (*p*_*cluster*_ = 0.02)	−3–8 29	0.15	13	0.14	0.01–0.47	17 ^S^
L parahippocampal (*p*_*cluster*_ = 0.04)	−22–21–18	0.25	23.4	0.22	0.008–0.74	31^VS^
R medial orbitofrontal (*p*_*cluster*_ = 0.00008)	7 39–11	0.17	15.1	0.11	0.0002–0.41	12508^E^
R superior parietal (*p*_*cluster*_ = 0.001)	4–62 7	0.13	10.5	0.10	0.0008–0.28	3.2 ^M^
R supramarginal (*p*_*cluster*_ = 0.01)	65–37 30	0.14	12.1	0.14	0.02–0.41	63^VS^
R posterior cingulate (*p*_*cluster*_ = 0.02)	6–8 42	0.20	18.2	0.22	0.02–0.73	100^E^

MNI coordinates are in MNI152 space. *f*^2^: measure of local effect size. CI_95%_: 95% confidence interval. BF_10_: Bayes factor derived from Bayesian linear regression analyses. The superscript of the BF_10_ indicates the qualitative interpretation of the evidence for the alternative hypothesis: ^M^ moderate; ^S^ strong; ^VS^ very strong; ^E^ extreme. L: left; R: right.

### Effects of global Aβ load on the relationship between T1w/T2w ratio and rs-FC

**Fig. 2:**
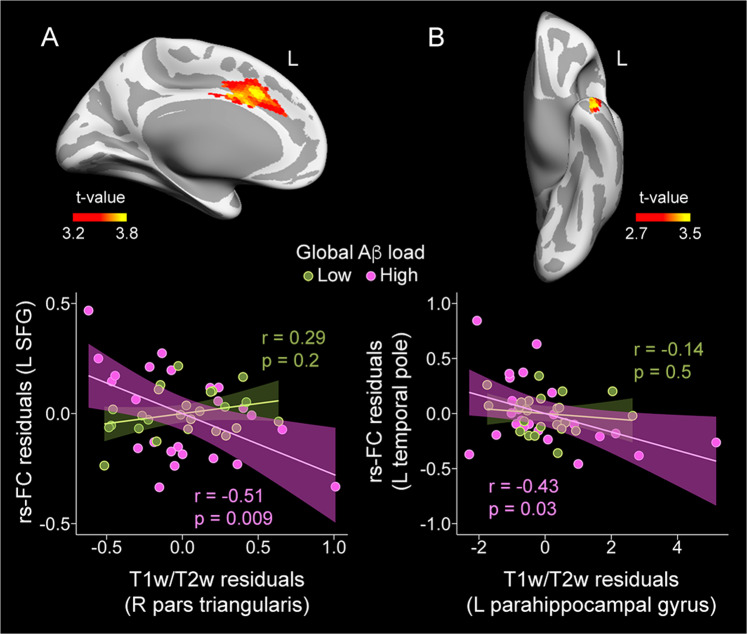
Partial regression plots showing the moderating effect of Aβ-related intracortical myelin changes on rs-FC networks. **A** Pattern of rs-FC using the right pars triangularis as FC seed (upper panel). Participants showing values 1 SD above the mean of global Aβ load (pink) revealed significant negative associations between T1w/T2w ratio in the right pars triangularis and rs-FC in the left superior frontal gyrus (SFG). **B** Abnormal pattern of rs-FC using the left parahippocampal gyrus as FC seed (upper panel). Participants showing values 1 SD above the mean of global Aβ load (pink) revealed significant negative associations between T1w/T2w ratio in the left parahippocampal gyrus and rs-FC in the left temporal pole. Figure axes show the residuals from the T1w/T2w ratio and the rs-FC.

We further assessed whether the strength of rs-FC shown by cortical regions exhibiting correlations between T1w/T2w ratio and global Aβ load differed as a function of global Aβ burden. Results of the frequentist and Bayesian approaches are summarized in Table 3. Multiple regression analyses revealed a moderating effect of global Aβ load for the association between T1w/T2w ratio in the right pars triangularis and the magnitude of rs-FC between this region and left superior frontal gyrus (Fig. 2, left upper panel). Post hoc analyses showed a negative association for participants showing global Aβ values 1 standard deviation (SD) above the mean while it was non-significant for those cases showing global Aβ values 1 SD below the mean (Fig. 2A, left bottom panel). A similar moderating effect was found for the association of T1w/T2w ratio in the left parahippocampal gyrus with rs-FC between this region and the left temporal pole (Fig. 2B, right upper panel). This significant association was negative for participants with higher global Aβ load and non-significant for the group with lower global Aβ load (Fig. 2B, right bottom panel).

**Table 3:** Effects of global Aβ load on the relationship between cortical myelin content and rs-FC.

*FC seed* Peak location of significant result	Extent of change (mm^2^)	MNI coordinates	F	P	*f*^2^
***R pars triangularis***					
L superior frontal gyrus	723	−10 20 35	14.4	10^–2^	0.2 _M_
***L parahippocampal***					
L temporal pole	243	−39 8–36	12.3	10^–2^	0.17 _M_

MNI coordinates correspond to MNI152 standard space. P: *p* value of the cluster; *f*^2^: measure of global effect size. Effect size (*f*^2^): small (S) ≥ 0.02, medium (M) ≥ 0.15, large (L) ≥ 0.35. L/R: left/right.

### Associations with cognition

We then investigated whether cognitive scores were associated with cortical regions affected by the relationship between global Aβ load and T1w/T2w ratio. The results of the frequentist and Bayesian approach are summarized in Table 4. Multiple regression analyses showed negative associations between general cognition (*g* factor), speed processing, attention and planning with T1w/T2w ratio in the posterior cingulate cortex (left or right hemisphere depending on the cognitive domain). Results further indicated a negative relationship between associative memory and T1w/T2w ratio in the left parahipocampal cortex. The largest effect sizes were observed for the *g* factor in the left posterior cingulate followed by speed processing in the same region and by associative memory in the left parahipocampal gyrus. Bayesian linear regression analyses showed extreme evidence for the negative correlations between the *g* factor and the posterior cingulate bilaterally (Table 4).

**Table 4:** Association of cognition with global Aβ load-induced changes in cortical myelin content.

	beta	F_1,66_	*p*	Size effect	CI_95%_	BF_10_
*g factor*						
L posterior cingulate	−0.78	27.9	10^–5^	0.24	0.02–0.72	180^E^
R posterior cingulate	−1.42	6.7	0.01	0.09	0.002–0.40	171^E^
*Associative memory*						
L parahippocampal	−1.78	6.3	0.01	0.13	0.01–0.37	8.8 ^M^
*Processing speed*						
L posterior cingulate	−0.93	9.8	0.002	0.19	0.02–0.55	56^VS^
R posterior cingulate	−2.25	4.2	0.04	0.09	0.001–0.57	3.7 ^M^
*Attention*						
R posterior cingulate	−1.28	7.6	0.007	0.11	0.007–0.33	7.3 ^M^
*Planning*						
L posterior cingulate	−0.46	5.5	0.02	0.08	0.001–0.30	3.3 ^M^

CI_95%_: 95% confidence interval. BF_10_: Bayes factor derived from Bayesian linear regression analyses. The superscript of the BF_10_ indicates the qualitative interpretation of the evidence for the alternative hypothesis: ^M^ moderate; ^S^ strong; ^VS^ very strong; ^E^ extreme. L: left; R: right.

The mediating role of global Aβ load was only evident for the association between T1w/T2w ratio in the left posterior cingulate cortex and processing speed. More specifically, causal mediation analysis showed significant regression coefficients between the T1w/T2w ratio of the left posterior cingulate and global Aβ load (0.15, *p* < 10^–5^) and between global Aβ load and processing speed (3.86, *p* = 0.017). The bootstrapped unstandardized indirect effect was 0.58, the 95% confidence interval ranged from 0.087 to 1.27, and the indirect effect was statistically significant (*p* = 0.02). However, the mediation was partial because the association between T1w/T2w ratio and processing speed remained different from zero when global Aβ load was introduced as a mediator.

Finally, we investigated whether the cognitive performance was also associated with patterns of rs-FC of cortical regions that previously showed a significant global Aβ load × T1w/T2w ratio. The Bayesian approach only revealed an association of mental flexibility with rs-FC between right pars triangularis and left superior frontal gyrus (BF_10_ = 3.84), which, however, could not be confirmed by the frequentist approach (F_1,65_ = 3.5, *p* = 0.06).

## Discussion

Accumulation and deposition of Aβ in the brain, considered as the key initial step in AD pathogenesis, is inevitable with advancing age. Approximately 25% of cognitively normal older individuals show evidence of Aβ pathology [1, 44, 45]. Therefore, determining the initial effects of Aβ burden, before cognitive decline occurs, may be crucial for early detection and treatment of AD. Regardless of whether Aβ burden precedes or results from myelin damage, evidence suggests that myelin breakdown is one of the earliest abnormalities contributing to synaptic dysfunctions before the onset of AD symptoms [46]. The present study contributes to strengthen this view. Here, we showed that increased global Aβ uptake was related to changes in cortical myelin content in cognitively normal older adults. Both decreases and increases in cortical myelination linked to Aβ pathology were further associated with altered patterns of rs-FC and subclinical cognitive deficits. Collectively, these findings provide an unprecedented opportunity for noninvasive evaluation of the association between Aβ burden and cortical myelin content in cognitively normal older adults.

The association between Aβ burden and intracortical myelin has been previously described in post-mortem AD brain tissue. These studies have shown that damage of cortical myelinated axons occurred concomitantly with the appearance of amyloid precursor protein (APP) and was accompanied by an increased expression of degraded myelin basic protein that co-localized with both myelin sheaths and Aβ plaques [47]. Our findings agree with previous research carried out in humans [48, 49] and transgenic mouse models of AD [11, 50, 51], indicating that initial Aβ deposition relates to changes in the microstructural integrity of cortical GM years before the hypothetical onset of clinical symptoms. It is important to emphasize that Aβ-PET retention has shown to be helpful for monitoring the progression of demyelination across the AD continuum [52] and multiple sclerosis [53], suggesting that Aβ-PET radiotracers bind WM myelin and therefore are able to indirectly relate to myelin content in vivo.

In line with our initial hypothesis, greater Aβ burden was found to be associated with lower myelin content in the left lingual gyrus, left anterior cingulate, and right aspects of the inferior frontal gyrus. These results are consistent with previous studies that highlight the vulnerability of these cortical regions to AD pathology and myelin damage. Accordingly, we have recently reported lower myelin content in the left lingual gyrus of asymptomatic older adults carrying the ɛ4 allele of the APOE gene [54]. This region has also shown lower strength of rs-FC in patients with mild cognitive impairment [55] and decreased activation while AD patients performed a memory task [56]. Similarly, higher Aβ retention in the anterior cingulate cortex has been associated with the development of preclinical AD [57]. Furthermore, recent research has revealed that greater global Aβ deposition is associated with higher activation of the right inferior frontal gyrus in cognitively normal older adults [58], supporting abnormal activation of this region in preclinical AD [59]. All these late-myelinating cortical regions are particularly susceptible to early AD pathology and, consequently, more prone to experience myelin breakdown [60]. Alternatively, Aβ pathology may result from the age-related increase in the need for myelin maintenance and repair, suggesting that Aβ burden is secondary to brain efforts to maintain myelin homeostasis [61].

Previous research has demonstrated that cerebral Aβ burden is also accompanied by an increase in oligodendrogenesis paralleled by thicker myelin sheaths and shorter nodes of Ranvier in transgenic mouse models of AD [13, 14]. These results are consistent with our data showing positive correlations between global Aβ load and cortical myelin content. Other studies have also shown abnormally higher myelination patterns in cortical regions that accumulate Aβ deposition before the onset of AD symptoms [62, 63]. These findings are interpreted as a compensatory remyelination in affected cortical regions [64], which may alter the speed of axonal conduction caused by shorter internodes of remyelinated axons [20, 65]. In line with this hypothesis, we found that global Aβ load partially mediated the negative association between myelin content in the left posterior cingulate gyrus and processing speed. These results support the idea that signaling pathways involved in both Aβ accumulation and myelin repair could contribute to deterioration in cognitive functioning [66].

Subtle changes in myelin thickness and/or internodal structure have profound effects on neuronal network function affecting spike-time arrival [20], which is of fundamental importance for neural coding, neuronal integration and synaptic plasticity [21]. Similarly, the frequency, propagation and coupling of intrinsic oscillatory neuronal activity within cortical networks is also strongly influenced by conduction delays and thus by myelin integrity of small-caliber cortical axons [67]. Consequently, conduction delays in myelinated cortical axons originated by external insults may ultimately lead to abnormal synchronization patterns of cortical networks. Consistent with these findings, older individuals with greater Aβ burden showed abnormal patterns of rs-FC of the pars triangularis and parahippocampal gyrus. However, abnormal patterns of rs-FC were not correlated with cognition, leading us to speculate that the negative impact of such functional reorganization may become evident when the cognitive decline begins and/or in presence of tau pathology.

Multiple lines of evidence support that altered myelination may be one of the mechanisms by which amyloid pathology leads to cognitive decline. First, Aβ deposits, which initially tend to spread into the neocortex^2^, may alter functional communication not only by disrupting intracellular calcium homeostasis and synaptic transmission in cortical networks [68, 69], but also by damaging myelinated cortical neurons, which are supposed to play a major role in synchronizing conduction velocity within cortical circuits [70]. Second, damage of myelinated cortical axons may also increase the metabolic requirements of cortical neurons to maintain neurotransmission levels [71]. This high-energy expenditure increases the production of free radicals, making oligodendrocytes especially vulnerable to oxidative insults [72]. This hypothesis is consistent with recent findings suggesting that oligodendrocytes are part of a neuronal antioxidant defense system to support iron detoxification [73]. Third, oligodendrocytes are not only responsible for axonal myelination but also for the synthesis of brain cholesterol [74]. About 70% of the cholesterol in the brain resides in axonal myelin sheaths, enabling the current to move down the axon rather than diffusing across the membrane [75]. The fact that aggregated Aβ shows high affinity for cholesterol binding in the cell membrane [76] could also lead to myelin damage and enhanced Aβ-induced toxicity in cortical networks [77]. The association between Aβ deposition and myelin breakdown could be bidirectional. Indeed, recent evidence has shown that myelin damage induced in mouse models of amyloidosis are also capable of driving Aβ deposition by shifting neuronal APP metabolism and by altering the microglial phenotype [17]. On the basis of these findings, we speculate that aging-dependent myelin loss may contribute to the appearance of initial Aβ deposits, whereas at later stages, Aβ aggregates may facilitate the downstream effects of Aβ and tau pathology in oligodendrocytes and their precursor cells.

This study has several limitations. First, changes in cortical T1w/T2w ratio maps could be influenced by factors other than demyelination [78, 79]. Accordingly, these results should be interpreted cautiously until the biological interpretation of cortical T1w/T2w ratio maps is more precise. On the other hand, these results are derived from a cross-sectional study. Therefore, they cannot shed light into how the relationship between global Aβ load and cortical myelination affects AD development or into the causal effects of Aβ burden on intracortical myelin and/or vice versa. Furthermore, the degree of AD pathology was only established for Aβ pathology. Further studies should assess if cortical myelin content is also related to the presence of abnormal tau biomarkers in cognitively normal older adults. Finally, our results were obtained with a small sample and therefore they should be considered as preliminary and replicated in further experiments with a larger and independent cohort.

In conclusion, we have shown that the global Aβ burden is related to changes in the cortical microstructure from very early on, likely affecting myelinated axons of cortical GM, as revealed by changes in cortical T1w/T2w ratio maps. Myelin alterations associated with Aβ pathology were further related to abnormal patterns of rs-FC and subclinical cognitive deficits. Although we are still far from understanding the role of myelin damage in aging and preclinical AD, these results reinforce the view that Aβ aggregates and intracortical myelin are coupled events that may accelerate cognitive decline, ultimately increasing the risk to develop AD.

## Data Availability

The datasets generated during the current study are available from the corresponding author on reasonable request.
